# Environmental influence on *Pristionchus pacificus* mouth form through different culture methods

**DOI:** 10.1038/s41598-017-07455-7

**Published:** 2017-08-03

**Authors:** Michael S. Werner, Bogdan Sieriebriennikov, Tobias Loschko, Suryesh Namdeo, Masa Lenuzzi, Mohannad Dardiry, Tess Renahan, Devansh Raj Sharma, Ralf J. Sommer

**Affiliations:** Department of Evolutionary Biology, Max Planck Institute for Developmental Biology, 72076 Tübingen, Germany

## Abstract

Environmental cues can impact development to elicit distinct phenotypes in the adult. The consequences of phenotypic plasticity can have profound effects on morphology, life cycle, and behavior to increase the fitness of the organism. The molecular mechanisms governing these interactions are beginning to be elucidated in a few cases, such as social insects. Nevertheless, there is a paucity of systems that are amenable to rigorous experimentation, preventing both detailed mechanistic insight and the establishment of a generalizable conceptual framework. The mouth dimorphism of the model nematode *Pristionchus pacificus* offers the rare opportunity to examine the genetics, genomics, and epigenetics of environmental influence on developmental plasticity. Yet there are currently no easily tunable environmental factors that affect mouth-form ratios and are scalable to large cultures required for molecular biology. Here we present a suite of culture conditions to toggle the mouth-form phenotype of *P*. *pacificus*. The effects are reversible, do not require the costly or labor-intensive synthesis of chemicals, and proceed through the same pathways previously examined from forward genetic screens. Different species of *Pristionchus* exhibit different responses to culture conditions, demonstrating unique gene-environment interactions, and providing an opportunity to study environmental influence on a macroevolutionary scale.

## Introduction

Phenotypes can be dramatically influenced by environmental conditions experienced during development, a phenomenon referred to as developmental plasticity^1–3^. Examples of plastic phenotypes have been studied for nearly a century, including differences in morphology^4^, sex and caste determination^5–7^, and innate immunity^8^. However, despite long-held interest in the field, and decade’s worth of progress linking genotype to phenotype, relatively little is known about the mechanisms connecting environment to phenotype. To study the mechanisms of environmental influence on phenotype, easily tunable methods to induce phenotypic changes and model organisms amenable to molecular biology techniques are required. For example, temperature and diet have been utilized to explore plasticity in insects and nematodes^9–14^, some of which have revealed fundamental principles of dynamic gene regulation. In particular, investigating life cycle plasticity in *C*. *elegans* contributed to our understanding of nutrition and endocrine signaling^15–18^, and the discovery of regulatory RNAs^19^. However, the number of case studies remains small, and heuristic insight of ecologically relevant phenotypes within an evolutionary framework is still lacking.

**Figure 1:**
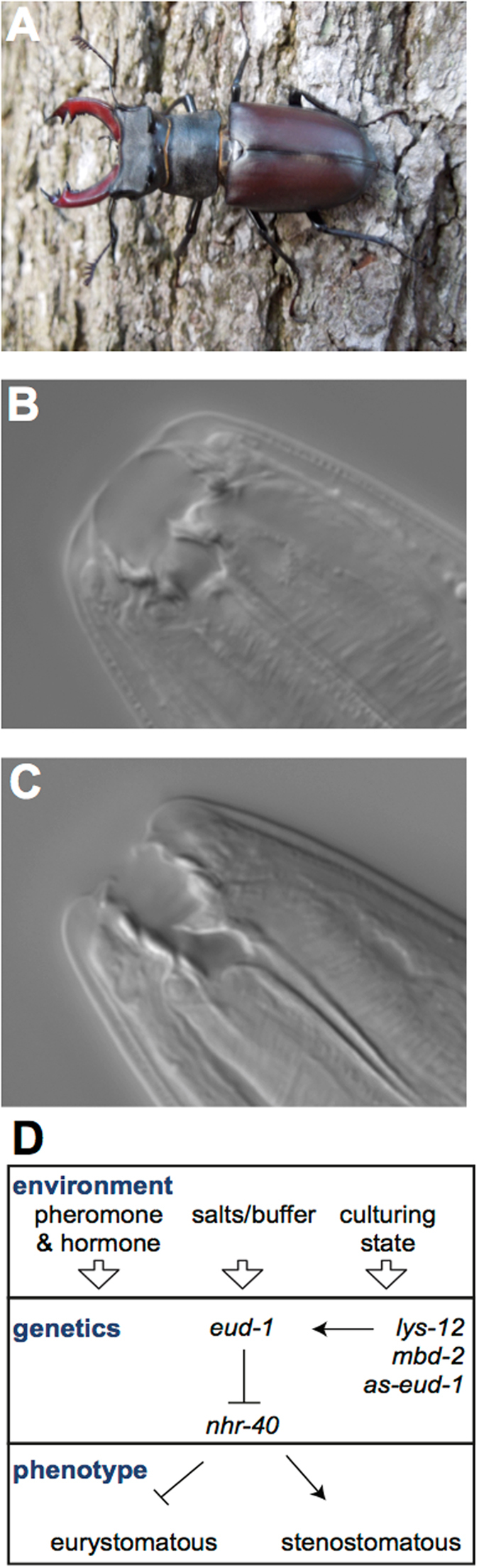
Life cycle and phenotypic plasticity of *Pristionchus pacificus*. (**A**) *P*. *pacificus* exist in a necromenic relationship with host beetles (i.e. shown here *Lucanus cervus*), and upon decomposition of the beetles the worms exit the dormant (dauer) state. Photo taken by M Herrmann and R Sommer. Depending on environmental conditions experienced during this period, adults develop either (**B**) a wide-mouth “eurystomatous” (Eu) morph with an additional tooth allowing them to prey on other nematodes, or (**C**) a microbivorous narrow mouth “stenostomatous” (St) morph. (**D**) Diagram integrating the environment into known gene-phenotype interactions of the *P*. *pacificus* mouth-form pathway.

The model organism *P*. *pacificus* exhibits an environmentally sensitive developmental switch of its feeding structures^20^. In the wild *P*. *pacificus* exists in a dormant state (dauer) on beetles. When beetles die *Pristionchus* exits the dauer state to feed on decomposition bacteria, and proceeds to reproductive maturity^21, 22^ (Fig. 1A). While developing under crowded conditions a “wide-mouthed” eurystomatous (Eu) morph with two teeth is built, which allows adults to prey on other nematodes (Fig. 1B). Alternatively, a “narrow-mouthed” stenostomatous (St) morph with one tooth relegates diet exclusively to microorganisms (Fig. 1C). While Eu animals can exploit additional food sources^23^ and attack and kill competitors^24^, St animals mature slightly faster^25^, creating a tradeoff of strategies depending on the environment perceived during development. Under monoxenic growth conditions in the laboratory using *Escherichia coli* OP50 bacteria as a food source on NGM-agar plates, 70–90% of the reference *P*. *pacificus* strain PS312 develop the Eu morph. Metabolic studies have elucidated compounds that affect this mouth-form decision. For example, the steroid hormone dafachronic acid shifts mouth-form frequencies to St^20^. Conversely, the pheromone dasc#1 shifts the frequency to Eu^26^. Recent mutant screens have established several genes in the mouth-form regulatory pathway^27–29^. The sulfatase *eud-1* (*eu*rystomatous *d*efective) is a dosage-dependent “switch” gene^30^: *eud-1* mutants are 100% St, while overexpression of a *eud-1* transgene confers 100% Eu^27^. The nuclear-hormone-receptor *Ppa-nhr-40* was identified as a suppressor of *eud-1*, and regulates downstream genes^28^. *C*. *elegans* homologs of the epigenetic enzymes acetyltransferase *lsy-12* and methyl-binding protein *mbd-2* have also been identified to control mouth-form plasticity, and are attractive factors for channeling environmental cues to changes in gene regulation. Both mutants led to global losses of activating-histone modifications, and decreased expression of *eud-1* ^29^.

Identification of these switch genes affords the opportunity to track regulatory mechanisms that respond to environmental cues^31, 32^. Unfortunately, the application of small molecules to affect mouth-form ratios in large enough quantities for biochemical fractionation or epigenetic profiling (e.g. ChIP) is impractical given the labor and expense of chemical synthesis or purification. Moreover, it is difficult to obtain consistent mouth-form ratios with pharmacological compounds as they are in constant competition with endogenous hormones and pheromones^20^. Finally, while crowding/starvation can also induce the Eu morph, it is technically challenging to compare different population densities, or to synchronize starved vs. un-starved larvae. To adequately study environmental effects on phenotypic plasticity, cheap, consistent, and simple methods are needed that can tune mouth-form ratios in synchronized populations. Here, we establish a set of culture conditions to affect environmental influence on mouth form. These methods are fast, reproducible, and only require the differential application of buffer, and culturing state (solid vs. liquid). Intriguingly, different species of *Pristionchus* exhibit different response regimes, suggesting evolutionary divergence of gene-environment interactions.

## Results

### Liquid culture affects *Pristionchus pacificus* mouth-form

**Figure 2:**
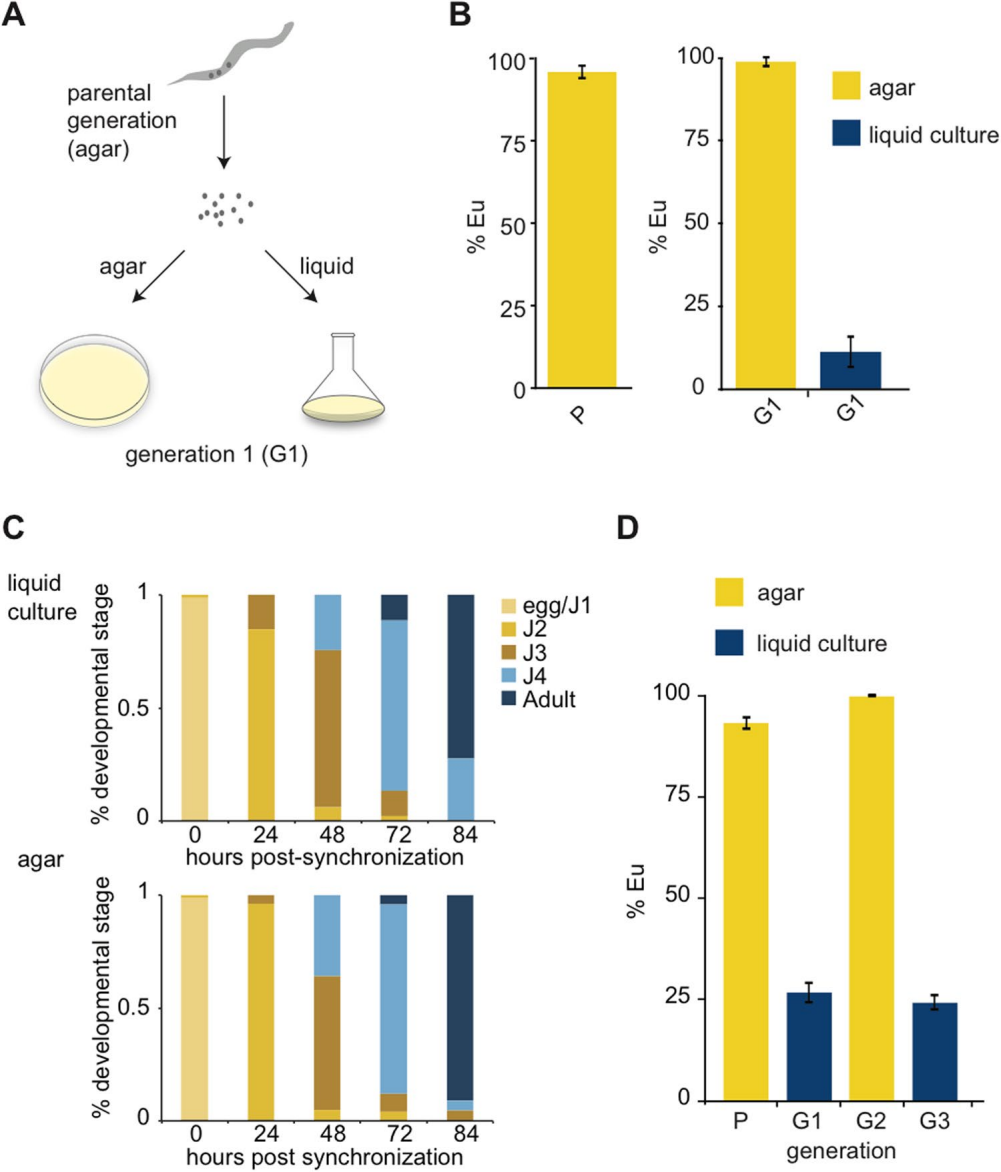
Different culture methods affect mouth-form phenotypic plasticity. (**A**) Diagram of experimental design to compare culture conditions from the same population after bleach synchronization. (**B**) Mouth-form ratios presented as percent eurystomatous (% Eu) from the parental generation (P) and the next generation (G1) grown in either liquid culture or agar plates, *n* = 18 biological replicates, *p* < 0.05, *students two-tailed t-test*, error bars represent SEM. (**C**) Developmental stages of bleach-synchronized *P*. *pacificus* in either agar plates or liquid culture. Bar graphs represent a typical experiment measuring >30 animals at the indicated time-points. (**D**) Mouth-form ratios of switching experiments between agar and liquid cultures. Nematodes were bleached between generations (P, G1, G2), and eggs-J1 larvae were passed to the next condition, *n* = 3, error bars represent SEM.

In order to accumulate large amounts of biological material for molecular and biochemical experiments we grew the laboratory California strain (PS312) of *P*. *pacificus* in liquid culture. To our surprise, this culture condition reversed the mouth-form phenotype from preferentially Eu to preferentially St. To better examine this observation we screened mouth-forms of adults representing a parental generation (P), and obtained^33^ and split eggs evenly to either agar plates or liquid culture, and screened adults of the next generation (G1) (Fig. 2A). Reproducibly, this simple difference in culturing method led to a dramatic shift in mouth-form ratio ( > 95% Eu on agar compared to ~10% Eu in liquid culture, *p* < 0.001, *paired t-test*) (Fig. 2B). Importantly, *P*. *pacificus* developed at similar rates in agar and liquid culture, allowing facile comparisons between conditions (Fig. 2C), and arguing against nutritional deprivation inducing the mouth-form shift. St animals have a slightly faster development than Eu animals when grown on agar^25^, however we found developmental speed to be indistinguishable between morphs in liquid culture (Supplementary Fig. 1). The different environmental conditions present distinct energy requirements (eg. swimming and feeding on motile bacteria in 3-dimensional liquid culture) that might offset potential tradeoffs in resource allocation.

Next, we investigated whether the change in mouth-form ratio induced by liquid culture was capable of being inherited. The mouth-from ratio of adults was consistent with the culture method they developed in regardless of the culture method of the parental generation, suggesting the effect is not transgenerational (Fig. 2D). These results also demonstrate the immediate and robust nature of this plasticity, and similar experiments coupled to mutagenesis may be useful for identifying genes involved in the ability to sense and respond to changing environments.

### Buffer components and culture state affect mouth form

To investigate the potential influence of culture conditions on mouth form we examined differences in buffer composition, and solid vs. liquid culturing state. In our previous experiments we had used standard liquid culture protocols for *C*. *elegans* ^33^, which utilize S media (S), whereas we normally grow *P*. *pacificus* on Nematode Growth Media (NGM) agar plates^33^. To assess the contribution of the chemical composition of the medium, as opposed to solid vs. liquid environments (hereafter referred to as ‘culture state’), we performed reciprocal culture experiments. Nematodes that were grown on either S-agar or NGM-liquid exhibited intermediate mouth-form ratios (51 ± 5% Eu and 38 ± 13% Eu, respectively, *p* < 0.001 relative to solid or liquid states of the same medium, *paired t-test*) (Table 1d,h,i,p), revealing a growth-medium composition effect. However, as these mouth-form ratios were in-between the extremes of NGM-agar and S-liquid, it also suggests other environmental factors are operating.

**Table 1:** Buffer/ions and physical culture state affect mouth-form phenotype.

	Condition	% Eu	S.E.M.
A	LC PBS, 180 rpm	7	3.2
B	LC Axenic Culture, 180 rpm	8.8	8.8
C	LC M9, 180 rpm	11.5	6.2
D	LC S-medium, 180 rpm	12.8	3.2
E	LC H-medium, 180 rpm	28	10.1
F	LC T-medium, 180 rpm	35	7.6
G	LC S-medium, 100 rpm	35.2	3
H	LC NGM, 180 rpm	37.8	12.9
I	AG S-medium	51.4	5.4
J	LC S-medium, 70 rpm	55.1	10.9
K	LC T-medium, 50 rpm	61.5	16.9
L	LC S-medium, 50 rpm	65.9	9
M	LC H-medium, 50 rpm	70.6	15
N	LC NGM, 50 rpm	87.3	3.3
O	NGG	97.1	2.5
P	AG NGM	98.7	0.7

A panel of culturing methods covers phenotypic ratios from ~10–99% Eu. LC = liquid culture, AG = agar, T and H medium = S-medium with phosphate replaced with 50 mM Tris or HEPES, pH 7.5, respectively, NGG = NGM with agar replaced with Gelrite/Gelzan CM (Sigma)^35^. *N* ≥ 3 biological replicates per condition, and standard error mean (SEM) is presented in the last column. Mouth-form phenotypes were assessed 4–5 days after bleach-synchronization (see Methods).

S medium contains phosphate (50 mM) and sulfate (14 mM) - both of which have previously been shown to affect mouth-form ratios at 120 mM^27^. To test whether this concentration of phosphate was causing the S-medium effect we made alternative formulations by replacing phosphate with 50 mM Tris (“T-Medium”) or Hepes (“H-Medium”), pH 7.5. Liquid culture in T- and H-medium yielded reproducibly higher Eu ratios (35 ± 8% and 28 ± 10%, respectively, p < 0.05, *paired t-test*) (Table 1d–f), demonstrating a specific, albeit subtle contribution from phosphate. Furthermore, *P*. *pacificus* grown in axenic (without bacteria)^34^, M9^33^, or PBS (which does not contain sulfate) -based liquid cultures were all highly St (Table 1a–c). Although nematode survival rate was poor in PBS, and development was slowed in axenic culture (9–10 days for sexual maturation, rather than 3–4).

### Rotation speed of liquid culture affects mouth form

Further exploration of liquid culture methodology revealed that decreasing the rotation per minute (rpm) also affected mouth-form ratios. Previous experiments that led to high St ratios had been performed at 180 rpm, but when shifted to “slow” speeds of 70 or 50 rpm, the mouth-form ratio shifted to an intermediate Eu bias (55 ± 11% and 66 ± 9%, respectively, *p* < 0.05, *t-test*) (Table 1j,l). The simplicity of changing rpm shaking-speed to affect mouth-form ratios is an intriguing environmental perturbation as other factors like food source, buffer, and culturing state are identical. When examined without bacteria, it became evident that at slow speeds (<90 rpm) nematodes aggregated in the center of the liquid column, whereas at higher speeds they were dispersed. When combined with conditions that exhibited intermediate St ratios the effects were additive, yielding up to 87 ± 3% Eu with NGM-liquid culture (Table 1k,m,n). The higher density of nematodes at slow speeds suggests that pheromones may be responsible. Consistent with this hypothesis, we passed multiple *P*. *pacificus* generations from one liquid culture to another, either by a 1:10 dilution, or by bleaching and washing. When passed by bleaching the next generation remained highly St (8 ± 4%). However when passed by dilution the next generation of worms exhibited intermediate Eu ratios (51 ± 16%, p < 0.05, *unpaired t-test*), perhaps because pheromones from the first generation were passed on to the second.

### Liquid culture affects body morphology

We also observed morphological differences of body length and width between agar and liquid culture, demonstrating an additional plastic response (Supplementary Figure 2). Worms that develop in liquid culture exhibit longer, narrower bodies compared to worms that develop in agar, a phenomenon that has also been observed in *C*. *elegans* ^33^. To disentangle whether the effect on mouth form is discrete or connected to the change in body shape we grew worms in NGG culture, which is intermediate between liquid and solid states^35^. Similar to liquid culture, adult worms grown in NGG exhibited a more slender body morphology than on agar plates (p < 0.05, Mann-Whitney), but they exhibited the highly Eu mouth-form ratio of worms grown in agar culture (Supplementary Fig. 2, Table 1d,o,p). While it is difficult to completely exclude the possibility that they are connected, there is no obvious correlation between the St mouth form and slender morphology observed in liquid culture. Therefore, it seems these two instances of phenotypic plasticity are under distinct regulation.

**Figure 3:**
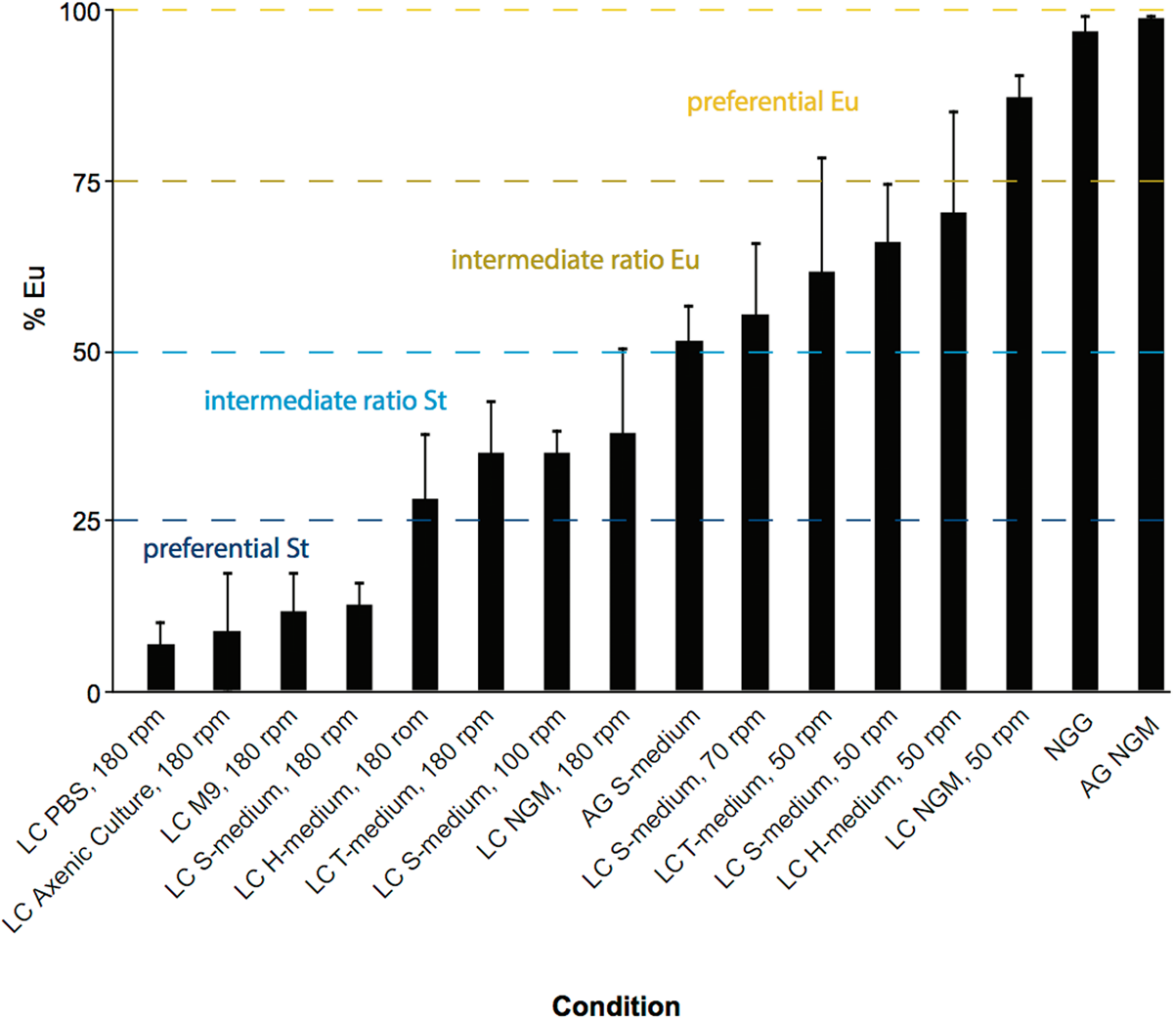
Comprehensive evaluation of culture method on mouth-form ratio in *P*. *pacificus*. Same data as in Table 1, but presented according to gradation of effect on mouth-form phenotype, from low to high % eurystomatous. LC = liquid culture, AG = agar, T and H medium = S-medium with phosphate replaced with 50 mM Tris or HEPES, pH 7.5, respectively, NGG = NGM with agar replaced with Gelrite/Gelzan CM (Sigma)^35^. Error bars represent standard error mean (SEM) for different biological replicates (*n* ≥ 3, Methods).

Collectively, we have established a broad range of culturing methods that allow the acquisition of almost any mouth-form ratio from an isogenic strain (Fig. 3). A variety of liquid culture conditions, including buffers without phosphates or sulfates, exhibited an effect on mouth form, suggesting an unknown environmental effect that is perhaps specific to solid or liquid states.

### Liquid culture acts upstream of known switch genes

**Figure 4:**
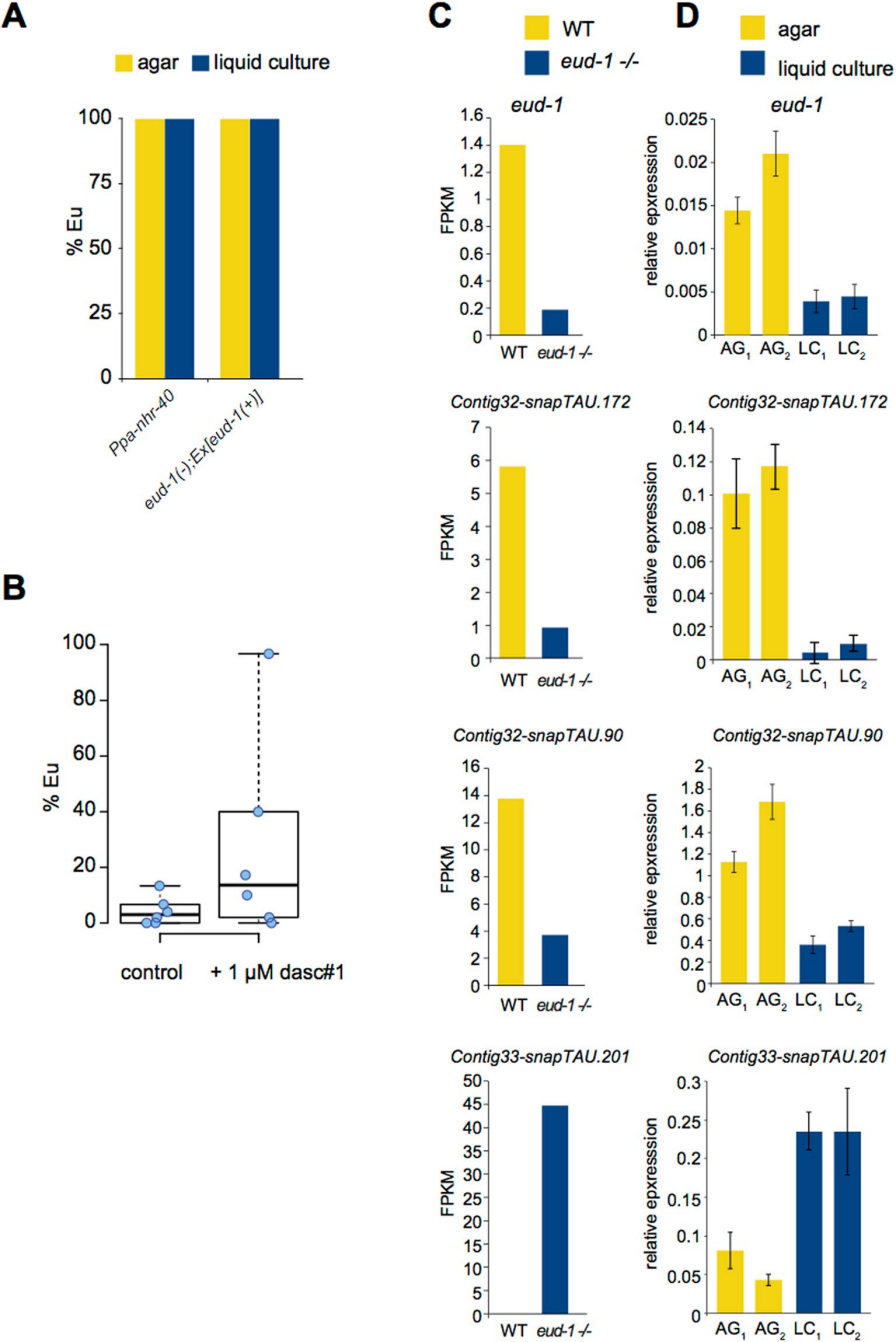
The environmental effect of liquid culture is upstream of known genetic components and induces similar pathways. (**A**) Mouth-form ratios of *eud-1* overexpression^27^ and a *Ppa-nhr-40* ^28^ mutant in liquid culture reveals no effects, suggesting these genes are downstream, *n* = 3 biological replicates. (**B**) Addition of 1 µM dasc#1 exhibits a variable response that appears to induce Eu, although it is not statistically significant (*p* = 0.068). (**C**) Expression analysis of four genes by RNA-seq from *eud-1* mutants (the average of 4 homozygous mutant alleles is represented)^27^ (100% St) compared to the RS2333 California strain (70–100% Eu), y-axis = fpkm (relative expression). (**D**) Reverse transcription-quantitative PCR (RT-qPCR) of *P*. *pacificus* PS312 grown in liquid culture/S-medium (LC) vs. NGM-agar plates (AG) for the two biological replicates displayed, with four technical replicates each. The y-axis represents 2^ΔCt^ (relative expression) compared to the housekeeping gene *Ppa-Y45F10D*.*4* (iron binding protein)^69^, error bars represent standard deviation of n = 4 technical replicates.

Next, we sought to place the environmental effects of liquid culture relative to known genetic and environmental factors. First, we examined whether liquid culture had an effect on mutants that are 100% Eu on agar plates^27, 28^. Animals from a *eud-1* overexpression line and *Ppa-nhr-40* mutant line remained 100% Eu in liquid culture, arguing that these genes act downstream of the environmental effect of liquid culture (Fig. 4A). Next, we assessed whether the dasc#1 pheromone was capable of inducing the Eu mouth-form in liquid culture, as it does on agar. dasc#1 experiments demonstrate a large variability in phenotypic ratio (Fig. 4B), however they typically exhibited a higher Eu proportion than control worms without dasc#1 treatment (*p* = 0.068, *paired t-test*). This intermediate and variable effect suggests that liquid culture and the dasc#1 pheromone act in parallel and antagonistically to each other. Finally, we also compared the expression of four genes in different culturing conditions that are up-or down-regulated in *eud-1* mutants (100% St) vs. wild-type (70–100% Eu)^27^. There was a strong correspondence between *eud-1* vs. wild-type RNA-seq data, and liquid vs. agar culture RT-qPCR (Fig. 4C,D). These results provide further evidence that the environmental effect of liquid culture is upstream of *eud-1*, and that this method is suitable for studying genetic pathways that have been determined through mutational experiments^27–29^.

### Liquid culture effect is dependent on genetic background

**Figure 5:**
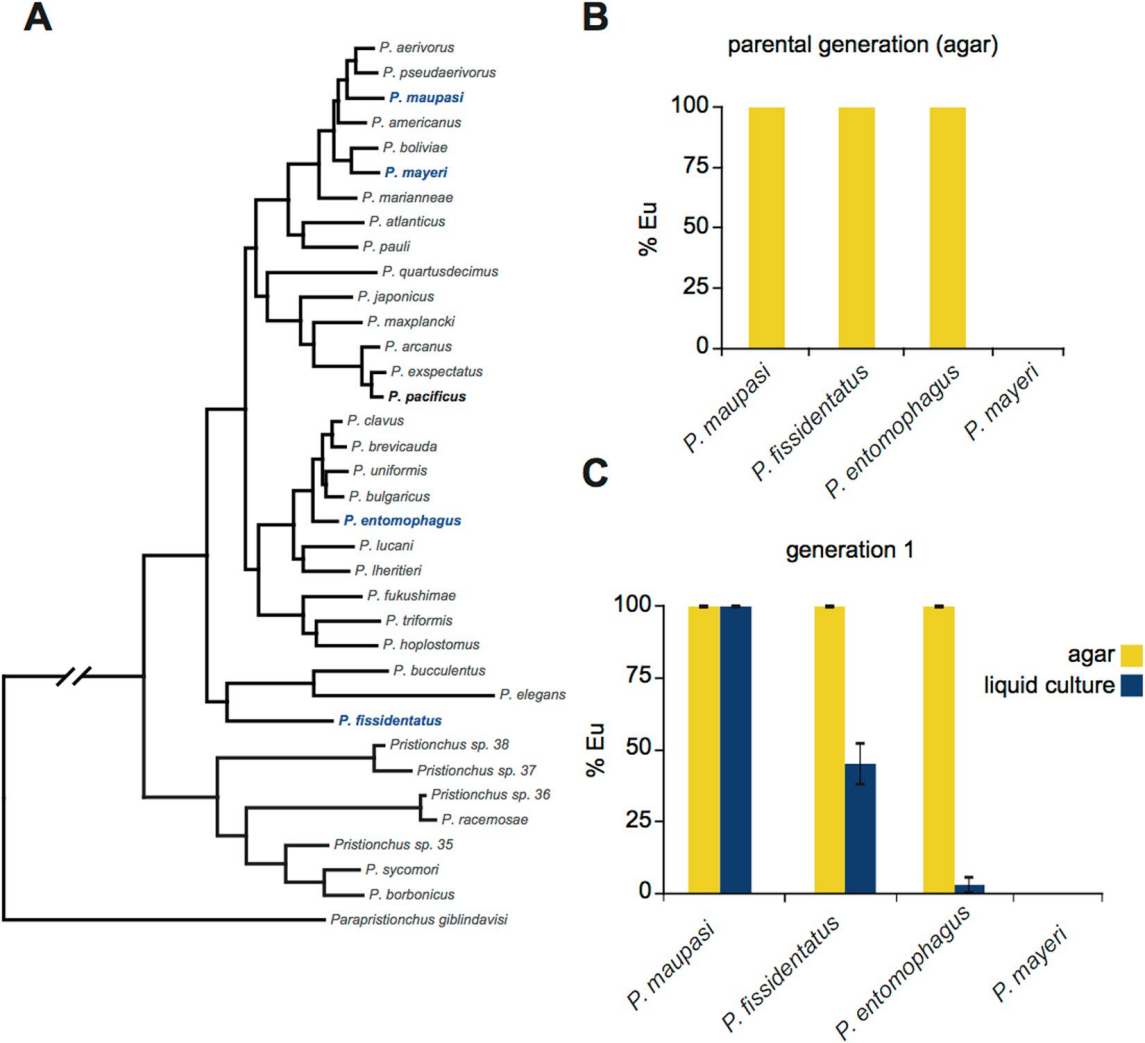
Macro-evolutionary view of liquid culture environmental influence. (**A**) Phylogeny of *Pristionchus* species^70^ highlighting *P*. *pacificus* (bold), *P*. *fissidentatus*, *P*. *mayeri*, *and P*. *entomophagus* (blue). (**B**) Mouth-form ratio of parental generations (*n = *3) of indicated species on NGM-agar after three consecutive healthy generations on OP50. (**C**) Mouth-form ratios of indicated species in either NGM-agar or liquid culture/S-medium (*n = *3), error bars represent SEM.

Finally, we explored whether there was a macro-evolutionary difference in responses to culture conditions. We chose four *Pristionchus* species that flank *P*. *pacificus* phylogenetically; three are highly Eu on agar (>95%), and one is highly St (>95%) (Fig. 5A,B). Remarkably, each species exhibited distinct phenotypic responses to liquid culture. For example, *P*. *maupasi* was highly Eu in both conditions, while *P*. *entomophagus* shifted to almost 100% St (Fig. 5C) in liquid culture. Meanwhile *P*. *mayeri* was St in both culture conditions. Taken together, these data show a genetic basis to environmental effects on phenotypic plasticity, which can be exploited for evolutionary, genetic, and molecular exploration of plasticity mechanisms. Whether these differences in response reflect adaptive changes to different environments, or are a result of drift remains to be seen in future investigations.

## Discussion

We describe multiple methods for the culture of preferentially St (<25% Eu), intermediate St (25–50% Eu), intermediate Eu (50–75 Eu%), and preferentially Eu (>75% Eu) *P*. *pacificus* (Fig. 3, Methods). Growth rates are similar between conditions, allowing the generation of developmentally synchronized populations. The effects are immediate, and immediately reversible when switching between liquid and agar, suggesting they are not transgenerational. Importantly, the genetic pathways towards building each respective mouth form are consistent with pathways established from prior forward genetics^27, 28^. Finally, the environmental response is unique in four species of *Pristionchus* tested, arguing that evolution has acted, passively or actively, on gene-environment interactions. The ability to toggle between mouth forms with simple culturing conditions provides powerful new tools to study the genetic and molecular mechanisms of phenotypic plasticity.

Perturbation of environmental factors such as salt concentration^15, 36–38^, pathogen^8, 39–42^, temperature^7, 10, 13, 43–45^, and diet^46, 47^ have been exploited for decades to study adaptive responses. More recent genome-wide profiling of epigenetic information carriers has revealed potential mechanisms for communicating stimuli to changes in gene expression. So called ‘poised’ or ‘permissive’ chromatin states can respond to external signals, leading to changes in transcription that ultimately affect tissue differentiation^48–55^. The time is now ripe to test whether similar processes affect phenotypic plasticity, a critical link between ecology and molecular mechanism that has just begun to be explored^56–60^.

Our panel of *P*. *pacificus* culture conditions saturates the mouth-form frequency space (Fig. 3). The ability to shift ratios by rpm shaking-speed provides perhaps the cleanest method because of its simplicity. In shaking speeds greater than 90 rpm nematodes are dispersed, while below 90 rpm they are concentrated in the center of the liquid vortex. Since different buffer formulations also affected mouth-form ratios, and the combination with slow rpm yielded an additive effect, it seems that alterations in the abundance, diffusion, and local concentration of pheromones and ions (i.e. phosphate and sulfate) contribute to the observed differences between liquid and agar culture conditions. However, we note that densely packed nematodes at slow rpm (much denser than on a plate) in NGM-liquid media are still insufficient to recapitulate the >95% Eu phenotype seen on NGM-agar plates. While it remains possible that these are the only contributing factors, we speculate an additional unknown factor is extant related to bacterial density, metabolism, or the liquid environment itself.

Whether liquid culture is a direct stimulator of the St mouth form is currently unknown. Field observations and competition experiments are required to (1) assess if *Pristionchus* experiences wet-enough conditions in the wild to mimic liquid culture conditions as with other lotic, lentic or marine nematodes^61–63^, and (2) determine whether the St mouth form provides an advantage in this environment. Both *C*. *elegans* and *P*. *pacificus* exhibit a slender morphology in liquid culture, suggesting a conserved plastic response to this environment. It is conceivable that a liquid culture-dependent signaling pathway related to mouth form also exists, although it could be mediated indirectly through other factors. Seemingly unrelated stimuli are capable of inducing the same developmental pathway by eventually descending on a downstream switch or “evocator”^64–66^. Regardless of the ultimate environmental factor, our analysis of gene expression in liquid culture reflects patterns observed in constitutive St mutants, suggesting that similar downstream pathways are utilized (Fig. 1D). Importantly however, we did not observe faster St development in liquid culture as has been observed on agar, and which is predicted to be the tradeoff advantage of the St morph^25^. It is formally possible that we did not have enough temporal resolution to identify the small but significant differences previously observed (55 hours for St and 61 hours for Eu). It is also worth noting that laboratory culture conditions are highly artificial, and it is perhaps not surprising that they could affect ecological strategies. Nevertheless, our results suggest that caution should be taken when studying *P*. *pacificus* ecology across different environments, as it may be context dependent. Going forward, it will be informative to assess if the developmental speed of different species correlates with their response to liquid culture, and the aqueous content in which they are found in nature.

Which culture method is utilized will depend on the purpose of the experiment. Exploiting intermediate ratio conditions may be useful to study genes or other environmental factors predicated to effect mouth form but in an unknown direction (Eu or St). For experiments that require the greatest separation in mouth-form frequencies we recommend S-medium at 180 rpm (St) vs. NGM agar plates (Eu). We also frequently observed a modest degree of variation, which is expected for a stochastic phenotypic trait^67^. As such, every measurement utilizing these culturing methods should be performed side-by-side with control samples. It is our hope that these methods will be a contribution to the study of environmental effects on *P*. *pacificus* mouth form, and phenotypic plasticity in general.

## Methods

### Strains and species

For all *P*. *pacificus* experiments the California strain PS312 was used, except comparisons to RNA-seq data, which used a more grown-out version of the same strain (RS2333). For experiments with different species (Fig. 5) *P*. *maupasi*, *P*. *fissidenatus*, and *P*. *mayeri* were compared to *P*. *pacificus*. Epistasis experiments (Fig. 4A) were performed with *Ppa-nhr-40*(*tu505*) and *eud-1*(*tu445*)*;tuEx[eud-1*(+)*]*.

### Culture methods

Five young adult *Pristionchus* nematodes were passed every 4–6 days on 10 ml NGM-agar, 60 mm plates at 20 °C seeded with 300 µl of overnight cultures of *Escherichia coli* OP50 (grown in LB at 37 °C) and covered with parafilm to avoid experiencing starvation for three consecutive generations^33^. The mouth-form phenotype of 4th generation adults represents the parental (P) generation (Fig. 2A,C, and D). Prior to all subsequent phenotyping experiments adults were synchronized by washing off of plates with M9 using plastic Pasteur pipettes into 15 ml conical tubes, and adding 30% final volume NaOH/bleach (0.5 ml NaOH, 1 ml bleach/3.5 ml washed worms) for 9 minutes with gentle agitation every few minutes. Carcasses were filtered through a 120 µm nylon net (Millipore) fixed between two rubber gaskets in a plastic funnel, washed by applying 3 ml M9 drop-wise on the filter, then pelleted 500 × g, 1 minute, room temperature. Eggs-J1 were washed by gentle re-suspension in 3 ml M9, and re-centrifuged 500 × g, 1 minute, room temperature. It is important not to wash worms with S-medium before or directly after bleach because it will start to precipitate. M9 wash was removed by pipette, and then eggs-J1s were ready for re-suspension in the appropriate buffer depending on the experiment.

For the majority of experiments, eggs-J1 larvae were re-suspended in 100 µl M9 × the number of test conditions (i.e. 200 µl for comparing one agar vs. one liquid culture condition). For re-culturing on agar, eggs-J1 were pipetted in the center of the OP50 lawn on 60 mm agar plates (NGM or S-medium), then the plate was tilted in 360° to spread and dry the eggs. Afterwards the plates were stored at 20° and adults were phenotyped 4–5 days later (see below for details of phenotyping). For culturing in liquid formats, 100 µl of eggs-J1 were pipetted into 10 ml of medium in 50 ml-volume autoclaved Erlenmeyer flasks. To prepare monoxenic liquid cultures the amount of OP50 *E*. *coli* was empirically determined. For all liquid cultures described (except axenic culture) 100 ml of overnight OP50 *E*. *coli* (grown in LB) to an optical density (OD_600_) of 0.5, was pelleted 30 minutes, 4 °C at 3,000 × g in an SLA-3000 rotor and re-suspended in 10 ml filter-sterilized (0.22 µm, Millipore) S-medium^33^ unless otherwise noted (e.g. M9 or PBS, Fig. 2). The concentration of bacteria is a critical parameter. The procedure described above led to healthy cultures of *P*. *pacificus* at the normal developmental rate observed on agar plates (3–4 days^21^), while adding less (50 ml or 10 ml) OP50 led to slower rates, or even the inability to develop beyond the J2 larval stage when significantly less was added. Liquid cultures were incubated 180 rpm, 20–22 °C unless otherwise noted for “slow” rpm experiments (50 and 70 rpm).

For experiments with “H” or “T” medium, S-medium was prepared as before^33^ except that phosphates were replaced with 50 mM of HEPES or Tris, pH 7.5, respectively. Axenic culture was prepared according to Samuel *et al*.^34^ with the exception that flavin-mononucleotide was replaced with riboflavin (Sigma) at the same amount, and cultures were shaken at 180 rpm instead of 70. As previously noted^34^ with *C*. *elegans*, *P*. *pacificus* also develops slower in axenic culture, reaching maturity (adults) at 9–10 days after adding eggs. Culture in NGG was performed similar to Muschiol and Traunspurger 2007^35^. In short, 3 ml of NGM was prepared with agar replaced with Gelrite/Gelzan CM (Sigma) at 0.75 g/L and seeded with 300 µl of OP50 and bleached eggs, then incubated at 20 °C.

To collect nematodes from liquid cultures for tracking developmental stages or mouth-form phenotyping we developed a filtering method using removable 5 µm filters (Millipore) combined with the Sterifil aseptic system (47 mm, Millipore). Filters are applied to the Sterifil apparatus and a small amount of M9 is added and vacuumed through to ensure a tight and continuous seal. Then liquid cultures are decanted into the funnel and slowly vacuumed. All *P*. *pacificus* developmental stages are large enough to be blocked by the 5 µm filter, while bacteria pass through. However when attempting to isolate J2s we recommend applying 2 × 5 µm filters. After all liquid has passed through the filter, nematodes were washed with ~25 ml of M9 by decanting directly on to the filter and applying vacuum pressure. Then the funnel was removed, and forceps were used to transfer the filter to an open 50 ml conical tube in a curved shape to fit into the opening. Nematodes were then washed from the filter by repeatedly applying the same 1 ml of M9 over the filter. Then this 1 ml was transferred to 1.5 ml microcentrifuge tube, and incubated at room temperature for 5 minutes to allow adults to swim to the bottom. Adults were pelleted by a quick (2–3 seconds) centrifugation, and the supernatant was removed. If juveniles are desired, the tube, now free of bacteria after filtering, can also be centrifuged at max speed >5 minutes to pellet. Nematode pellets were then phenotyped, or flash-frozen in liquid N2 and stored −80 °C for subsequent processing (e.g. RT-qPCR).

### Developmental rate determination

Worms were grown in liquid culture after bleach synchronization then filtered through a 20 µM filter 2 hours post bleach to isolate synchronous J2 animals, and then returned to liquid culture. Individual aliquots from the same flasks were monitored at regular intervals, and mouth-forms of adults were recorded at the J4-adult transition (*n* = 2). Flasks were rotated at 50 rpm to obtain large quantities of both St and Eu animals. Although not shown, several J4 were present at the earlier time points of 59 and 62 hours, which verified that we were observing the J4-adult transition.

### Mouth-form phenotyping

For phenotyping nematodes grown on agar plates or NGG^35^, adults were selected with a wire pick and transferred to 3–5 µl of M9 spotted on 4% agar pads (containing 10 mM sodium azide) on a standard microscope slide, then covered with a cover slip. For nematodes grown in liquid culture, after gently pelleting adults, they were re-suspended in the remaining M9 and 3–5 µl were directly pipetted onto the agar pad. When comparing mouth-forms of different conditions, we often performed ‘blind’ comparisons by writing the identity of the sample (i.e. “agar” or “liquid”) on the slide, and then using laboratory tape to cover the identity, and blindly selecting a slide before placing it in the microscope holder. After counting, the identity of the sample was revealed by removing the tape. Phenotyping was performed at 40–100x/1.4 oil objective on a Differential Interference Contrast (DIC) microscope (Zeiss) according to buccal landmarks previously described^20^. In short, Eu were determined by the presence of a wide-mouth, a hooked dorsal tooth, and an additional subventral tooth. Conversely St animals were determined by a narrow-mouth, flint-like dorsal tooth, and absence of a subventral tooth (Fig. 1B,C). The number of biological replicates (*n*) was ≥3 for all conditions, and as high as 18 for liquid culture/S-medium, with each replicate including ≥50 animals with the exception that PBS and NGM-liquid cultures yielded significantly fewer animals, and included ≥20 animals per replicate. Mouth-forms were assessed 4–5 days after bleach-synchronization. Error bars represent standard error means (SEM), and statistical significance was assessed by *paired 2-tailed t-tests* unless otherwise indicated in the text.

### dasc#1 experiments

dasc#1 was added at 1 µM final concentration according to previous methods^26^ to eggs-J1 larvae in liquid culture. Mouth-forms were phenotyped as described above after 4 days and compared to control liquid cultures without dasc#1. The p-value was determined by a 1-tailed, paired *t-test* for *n* = 6 biological replicates.

### Morphology measurements

Length and width measurements were performed on synchronized adult animals four days after bleaching. Measurements were made of 12 animals grown on agar, 13 grown on NGG, and 10 in liquid culture using the ImageJ plug-in WormSizer^68^. Box plots in Supplementary Figure 2 show quartile edges (25% and 75%) of the distribution and medians (black bars), made in R {boxplot(shape~Condition, data = worm_sizes, horizontal = TRUE, notch = FALSE)}.

### Expression analysis

RNA-seq data was obtained from Ragsdale, Müller *et al*.^27^, and average fpkms from 4 mutant alleles of *eud-1* vs. one wild-type California RS2333 were plotted. For RT-qPCR, RNA was first extracted from either 1 agar plate or 1 liquid culture of synchronized young adults (4 days post-bleaching) of the California strain PS312 (same as RS2333 but an earlier frozen stock) by Trizole extraction followed by purification with Zymo RNA-Clean & Concentrator-25 columns following manufacturers instructions from Zymo. 500–1,000 ng of purified RNA was converted to cDNA using SuperScript II (Invitrogen) for 1 hour with Oligo(dT)_18_ primer in 20 µl reactions, and then heat-inactivated with 40 µl of 150 mM KOH/20 mM Tris-base for 10 minutes at 99 °C followed by 40 µl of 150 mM HCl, and 100 µl of TE. 4 µl of cDNA was used for each technical replicate in 10 µl qPCR reactions with 1x LightCycler^®^ 480 SYBR Green I Master Mix (Roche) and 0.25 µM of each primer on a Light-Cycler 480, 384 well format. All primer sets were validated for single amplicon production with Tm melt-curve analysis, and efficiency with a 5-log titration of cDNA. Relative expression (2^ΔCt^) was measured relative to *Ppa-Y45F10D*.*4* (iron binding protein)^69^ for each gene.

### Data availability

All data generated or analyzed during this study are included in this article and its Supplementary Information files.

## Electronic supplementary material

Supplementary Information
