# Supplementary material for: Environmental influence on Pristionchus pacificus mouth form through different culture methods
Source: Sci Rep. 2017 Aug 3;7:7207. doi: 10.1038/s41598-017-07455-7 (PMC5543044; doi:10.1038/s41598-017-07455-7)
Supplement: Supplementary file 1 — Supplementary Information [file 41598_2017_7455_MOESM1_ESM.pdf]

*Supplementary Information*

# Environmental influence on *Pristionchus pacificus* mouth form through different culture methods

Michael S. Werner, Bogdan Sieriebriennikov, Tobias Loschko, Suryesh Namdeo, Masa Lenuzzi, Mohannad Dardiry, Tess Renahan, Devansh Raj Sharma and Ralf J. Sommer*

[1]Department of Evolutionary Biology, Max Planck Institute for Developmental Biology, 72076 Tübingen, Germany

*Correspondence: ralf.sommer@tuebingen.mpg.de

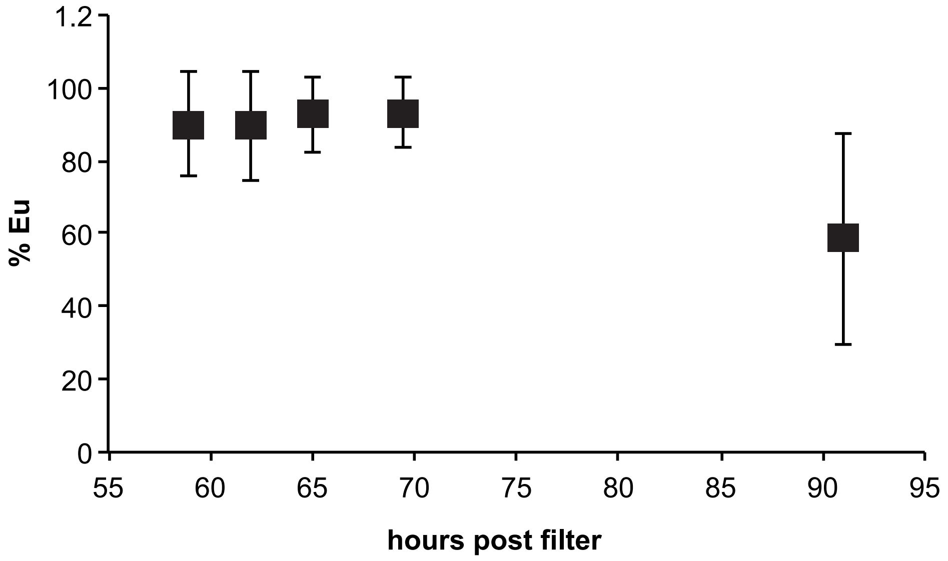


**Supplementary Figure 1. Growth rate of morphs in liquid culture.** Percent Eu of adult hermaphrodites grown in liquid at the J4-adult transition (59-70 hours post filter), and at 91 hours, a time point at which we normally collect and phenotype animals (n = 2). Worms were incubated at 22° C in S-Medium with 50 rpm shaking to induce sufficient numbers of both, St and Eu animals, allowing statistical significance testing ($p > 0.05$ between any two time-points arguing against slower development of Eu animals, two-tailed t-test).

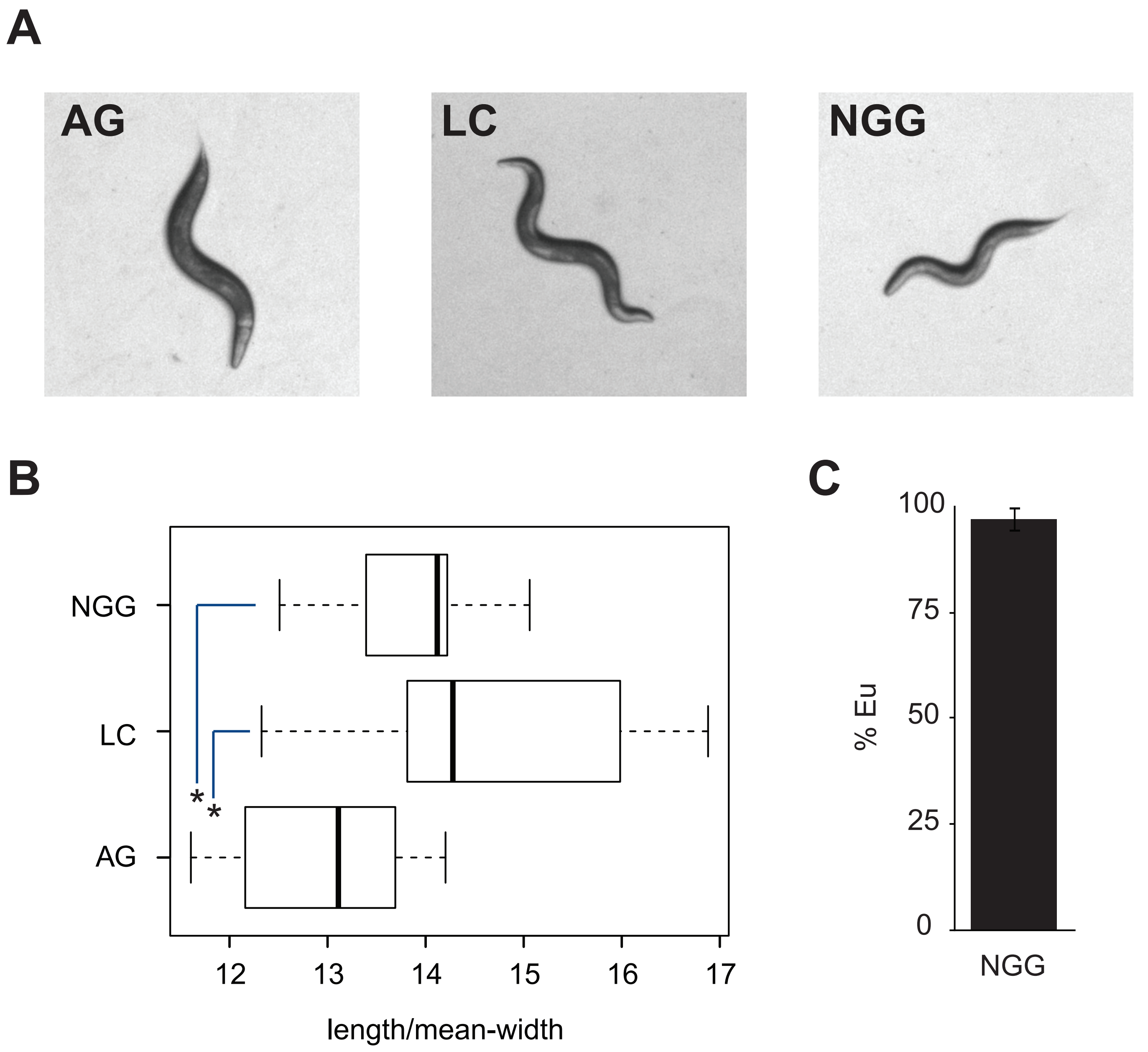


**Supplementary Figure 2. Slender morphology does not correlate with mouth-form.** (A) Images of *P. pacificus* grown in liquid culture and NGG display more slender morphology than on agar plates, quantified in (**B**). Measurements of adults from the same synchronized population were made with Wormsizer[68], n = 12 (agar), 13 (NGG), and 10 (liquid culture = 'LC'). Statistical significance was measured with a nonparametric Mann-Whitney U test in R. (**C**) Same as in Figure 2, mouth-form ratio of adult PS312 grown in NGG, n = 3.
